# Mitochondrial relationships between various chamomile accessions

**DOI:** 10.1007/s13353-020-00602-3

**Published:** 2020-12-08

**Authors:** Joana Ruzicka, Marion Hacek, Johannes Novak

**Affiliations:** Institute of Animal Nutrition and Functional Plant Compounds, University of Veterinary Medicine Vienna, Veterinaerplatz 1, A-1210 Vienna, Austria

**Keywords:** Mitochondrial genome, High resolution melting (HRM), Single nucleotide polymorphism (SNP), Asteraceae, *Matricaria chamomilla*

## Abstract

*Matricaria chamomilla* L. (GRIN; The Plant List 2013) is an important medicinal plant and one of the most frequently consumed tea plants. In order to assess mitochondrial genome variation of different cultivated chamomile accessions, 36 mitochondrial SNP markers were used in a HRM (high resolution melting) approach. In thirteen accessions of chamomile (*n* = 155), twenty mitochondrial haplotypes (genetic distances 0.028–0.693) were identified. Three of the accessions (‘Camoflora’, ‘Mat19’ and ‘Manzana’) were monomorphic. The highest genotypic variability was found for the Croatian accession ‘PG029’ with nine mitochondrial haplotypes (mitotypes) and the Argentinian ‘Argenmilla’ with seven mitotypes. However, most of the mitotypes detected in these accessions were infrequent in our sample set, thus disclosing an unusual high amount of substitutions within the mitochondrial genome of these accessions. The mitotypes with the highest frequency in the examined dataset were MT1 (*n* = 27), MT9 (*n* = 23) and MT17 (*n* = 20). All of the frequent mitochondrial lines are distributed not only over several accessions but also over several geographical origins. The origins often build a triplet with on average two to three concurrent lines. The most distantly related accessions were ‘Mat19’ and ‘Camoflora’ (0.539), while ‘PNOS’ and ‘Margaritar’ (0.007) showed the lowest genetic distance.

**Supplementary Information:**

The online version contains supplementary material available at 10.1007/s13353-020-00602-3.

## Introduction

*Matricaria chamomilla* L. (common name German chamomile, Asteraceae) (Germplasm Resources Information Network (GRIN) 2019; The Plant List 2013) is a mainly outcrossing and originally diploid (2n = 18) species indigenous to Europe and West Asia with natural populations occurring in many temperate regions worldwide (Franke and Schilcher 2007). More than 120 constituents have been identified in the chamomile flowers, out of which the majority is represented by the terpenoids α-bisabolol and its oxides (≤ 78%) and azulenes (1–15%) (Gupta et al. 2010). The high consumption and increasing market demand necessitated directed breeding concepts to improve crop yields, essential oil contents, pick height and disease resistance (Das 2014; Albrecht et al. 2016). Chamomile breeding started approximately 70 years ago and included diploid as well as tetraploid varieties artificially induced by colchicine treatment (Franke and Schilcher 2007). The Czech and Slovak Republic, Poland, Hungary and Germany were the nations in which the first breeding activities were located (Das 2014; Seidler-Lozykowska 2016). However, information about the natural populations serving as origins is scarce. The use of molecular approaches could help to resolve the genetic relationship between different chamomile varieties. Several molecular approaches to maintain breeding processes were conducted in the past (Wagner et al. 2005; Solouki et al. 2008; Pirkhezri et al. 2010; Okon et al. 2013; Ahmadi et al. 2014). SNP (single nucleotide polymorphism) analyses (Otto et al. 2017) are still rare, though, and genetic diversity studies mostly relied on nuclear polymorphisms. The exploitation of the mitochondrial (mt) diversity of *M. chamomilla* L. is just at the beginning. Uniparentally inherited markers of the chloroplast or mitochondrial genome proved adequate to enable investigations of migration routes of populations or the exploration of geographical backgrounds and common ancestors that date back several years (Tomaru et al. 1998; Gugerli et al. 2001; Arroyo-García et al. 2006). Information about mt genome diversity bears another advantage. Crossings of plants with highly variable or distantly related cytoplasm might enhance the possibility to find CMS (cytoplasmic male sterility) in the progenies (Spassova et al. 1993). CMS results from a conflict between nuclear and cytoplasmic genomes (Spassova et al. 1993; Budar et al. 2003). This sterility trait is widely used in breeding concepts of crop plants to assure controlled cross-pollination and prevent self-fertilization (Balk and Leaver 2001; Liu et al. 2013, 2014). For chamomile cultivation, where self-fertilization and seed-shedding is a serious problem, the establishment of a male sterile mother line would be of enormous advantage. Increased male sterility in the progenies of inter-cultivar crosses (Faehnrich et al. 2013) is already documented for chamomile but a CMS phenotype was not yet exhibited.

High-throughput technologies like GBS (genotyping by sequencing) and next-generation sequencing (NGS) were used recently to appraise genetic diversity of different chamomile accessions by exploiting a multitude of SNP data of the nuclear and mt genome (Otto et al. 2017; Ruzicka and Novak 2020). The mt sequence information of 33 chamomile individuals published by Ruzicka and Novak (2020) now enables the analysis of point mutations in a broader sample set using a high-throughput technology like HRM (high resolution melting). HRM is a very sensitive ‘post-PCR’ method, which relies on the different melting behaviours of double-stranded DNA fragments due to their varying sequence and GC content, and already has a fix position in SNP detection, e.g. for plant breeding and genotyping (Borna et al. 2017; Sorkheh et al. 2017; Kim and Kim 2019).

This study should proceed examining the mt diversity of different chamomile accessions for (I) reconstructing their breeding history and (II) estimate diversity between/among accessions and therefore test the applicability of the SNP markers in terms of distant inter-cultivar crossings.

## Material and methods

### Plant material

Thirteen accessions of *Matricaria chamomilla* L. were cultivated in the greenhouse at the University of Veterinary Medicine, Vienna (48° 15′ N, 16° 25′ E, 161 m a.s.l.), in 2017. Five, ten or 15 individuals per accession (Table 1) were harvested in June 2017 and used for SNP screening. Main ploidy levels as declared by Das (2014), Otto et al. (2017) and Faehnrich et al. (2019) were assumed for the accessions and were not further verified in the present study.

**Table 1:** Source, origins, number of individuals and declared ploidy levels (according to Das 2014, Otto et al. 2017 and Faehnrich et al. 2019) of the accessions used in this study

Accession	Ploidy level	Number	Origin	Source
Goral	4×	15	Slovak Republic	Pharmaplant Arznei-und Gewürzpflanzen Forschungs-und Saatzucht GmbH, Artern, Germany
Margaritar	4×	10	Romania	Pharmaplant Arznei-und Gewürzpflanzen Forschungs-und Saatzucht GmbH, Artern, Germany
PNOS	4×	15	Poland	Pharmaplant Arznei-und Gewürzpflanzen Forschungs-und Saatzucht GmbH, Artern, Germany
Manzana	4×	10	Austria	Ina Bein-Lobmaier, Versuchswirtschaft Groß-Enzersdorf, Austria
Mat90	4×	10	Germany	Pharmaplant Arznei-und Gewürzpflanzen Forschungs-und Saatzucht GmbH, Artern, Germany
Argenmilla	2×	15	Argentina	Pharmaplant Arznei-und Gewürzpflanzen Forschungs-und Saatzucht GmbH, Artern, Germany
Promyk	2×	10	Poland	Pharmaplant Arznei-und Gewürzpflanzen Forschungs-und Saatzucht GmbH, Artern, Germany
Camoflora	2×	15	Germany	Pharmaplant Arznei-und Gewürzpflanzen Forschungs-und Saatzucht GmbH, Artern, Germany
PG029	2×	15	Croatia	Pharmaplant Arznei-und Gewürzpflanzen Forschungs-und Saatzucht GmbH, Artern, Germany
MAT 16	2×	15	Bulgaria	Genebank Gatersleben, Germany
MAT 19	2×	15	North Korea	Genebank Gatersleben, Germany
Bona	2×	5	Slovak Republic	Company Vilora, Stará Ľubovňa, Slovak Republic
Soroksári 40	2×	5	Hungary	Corvinus University of Budapest, Faculty of Horticultural Sciences, Hungary

### DNA extraction

Approximately 1 cm^2^ of dried leaf material was ground with glass beads in a swing mill (Mixer Mill MM301, Retsch GmbH, Germany), and total genomic DNA was isolated using a modified CTAB (cetyltrimethylammonium bromide) extraction protocol (Schmiderer et al. 2013). Quantity and quality of the DNA were measured on a spectrophotometer (NanoDrop™ 2000, Thermo Fisher Scientific Inc., USA) and via gel electrophoresis on a 1.4% agarose gel stained with peqgreen (VWR, Austria). The DNA was dissolved in TE (Tris-EDTA, pH 8) buffer and stored at − 20 °C until further usage.

### SNP detection and primer design

NGS sequence information of 33 chamomile samples from eleven accessions was elaborated in a previous study (NCBI, National Center for Biotechnology Information, https://www.ncbi.nlm.nih.gov/, Submission ID: SUB5046906/BioProject ID: PRJNA515664). A LASTZ alignment of the resulting individual mt consensus sequences and *Diplostephium hartwegii* (KX063855) as reference sample generated with Geneious 9.1.5 (http://www.geneious.com, Kearse et al. 2012) was used for SNP detection and primer design. SNPs were detected using the SNP caller as implemented in Geneious under default parameters.

All primers were designed in Geneious (Version 9.1.5) (http://www.geneious.com, Kearse et al. 2012) with the primer feature that is based on the program Primer3 (Koressaar and Remm 2007; Untergasser et al. 2012) and were synthesised by Sigma-Aldrich Handels GmbH (Austria) (Online Resource 1).

### HRM analysis

HRM data were generated with the Rotor-Gene™ 6000 and the Rotor-Gene Q Series Software 2.1.0 (Qiagen, Germany).

The final reaction volume of 10 μl contained 1× HRM mastermix (HOT FIREPol® EvaGreen® HRM Mix, Solis BioDyne, Estonia), 150 nM of the forward and reverse primer each, and 2 ng genomic DNA. All samples and ‘no template controls’ (NTC) were analysed in duplicates.

The HRM analysis with pre-amplification was performed with an initial phase of 14 min at 95 °C, 45 cycles of 95 °C/ annealing temperature depending on the primer pair (Online Resource 1)/72 °C for 10 s/ 20 s/ 20 s. After PCR, a hold of 95 °C/1 min was implemented for complete denaturing of all nucleotide strands. HRM was done with a ramp of ~ 10 °C around the melting point of the synthesized DNA fragment (Online Resource 1) and in increments of 0.1 °C and 1 s hold before the temperature increase.

### Evaluation of the HRM analysis

The experimental work was performed according to the MIQE (Minimum Information for Publication of Quantitative Real-Time PCR-Experiments) guidelines (Bustin et al. 2009) wherever applicable. qPCR results were checked for average fluorescence level and average C_q_ value of the samples. Products with C_q_ values over 30 and under 17 and those with an end point fluorescence level of less than 80% of the average were excluded from the analysis and repeated in a later run. For the HRM analysis, the normalised and temperature shifted melting curves were used for the evaluation of the samples. Previously sequenced samples served as reference samples for the different curve types in the HRM analysis. These references were used to automatically assign the unknown samples to predefined genotypes by the Rotorgene software and were added to each run to check for interrun comparability. For the automated classification of the samples by the software, a confidence level of 70% was set. However, most of the samples were anyway correlated with a confidence of above 90%. Outliers were omitted and repeated. For each run, a clear clustering of the curve types in the fluorescence versus temperature plot was obligatory.

For the primer set Mr_mt130788 that detected a triallele (A/C/T), artificial heteroduplexes had to be produced to separate the melting curves of alleles A and T. Therefore, a spike DNA with the known haplotype A was added to the mastermix, resulting in identical HRM curves and melt curves with only one peak for samples with the haplotype A and heteroduplexes and two peaks in the melt analysis for haplotype T samples.

The mean standard deviation of T_m_ per run and ΔT_m_ were calculated for each primer combination (Table 2).

**Table 2:** Characteristics of the SNP markers used in this study. Numbers in locus names indicate positions of the substitutions in the alignment, SD refers to a mean within-run std.dev. of melting points of the curve types, ΔT_m_ to melting curve differences between the curve types as determined by HRM

Locus	No. of observed alleles	ΔT_m_	SD	λ	H_exp_	E.5
Mr_mt006392	2	0.37	0.02	0.132	0.133	0.520
Mr_mt006636	2	0.59	0.02	0.492	0.496	0.985
Mr_mt015448	2	0.43	0.01	0.489	0.492	0.978
Mr_mt016153	2	0.66	0.04	0.175	0.176	0.566
Mr_mt019240	2	0.40	0.03	0.489	0.492	0.978
Mr_mt026887	2	0.32	0.02	0.234	0.236	0.628
Mr_mt029259	2	0.60	0.02	0.234	0.236	0.628
Mr_mt035773	2	0.58	0.03	0.234	0.236	0.628
Mr_mt036292	2	0.34	0.02	0.288	0.290	0.687
Mr_mt038303	2	0.48	0.03	0.253	0.254	0.648
Mr_mt046728	2	0.49	0.05	0.025	0.026	0.366
Mr_mt071402	2	0.39	0.03	0.489	0.492	0.978
Mr_mt072537	2	0.59	0.04	0.489	0.492	0.978
Mr_mt073689	2	0.29	0.01	0.234	0.236	0.628
Mr_mt073751	2	0.80	0.02	0.164	0.165	0.555
Mr_mt085333	2	0.38	0.02	0.489	0.492	0.978
Mr_mt086300	2	0.50	0.03	0.038	0.038	0.394
Mr_mt092081	2	0.38	0.02	0.225	0.226	0.618
Mr_mt094432	2	0.51	0.02	0.038	0.038	0.394
Mr_mt101294	2	0.41	0.02	0.492	0.496	0.985
Mr_mt112276	2	0.47	0.02	0.050	0.051	0.416
Mr_mt130788	3	0.38	0.02	0.612	0.616	0.912
Mr_mt142266	2	0.34	0.02	0.477	0.480	0.956
Mr_mt143059	2	0.39	0.03	0.253	0.254	0.648
Mr_mt154969	2	0.25	0.03	0.013	0.013	0.327
Mr_mt158770	2	0.34	0.03	0.154	0.155	0.544
Mr_mt158868	2	0.82	0.05	0.025	0.026	0.366
Mr_mt187230	2	0.32	0.02	0.304	0.306	0.706
Mr_mt208653	2	0.38	0.02	0.492	0.496	0.985
Mr_mt233663	2	0.49	0.02	0.492	0.496	0.985
Mr_mt239816	2	0.51	0.03	0.143	0.144	0.532
Mr_mt239853	2	0.43	0.02	0.050	0.051	0.416
Mr_mt244062	2	0.45	0.01	0.304	0.306	0.706
Mr_mt262199	2	0.36	0.02	0.225	0.226	0.618
Mr_mt264622	2	0.46	0.03	0.050	0.051	0.416
Mr_mt277601	2	0.37	0.02	0.288	0.290	0.687
**mean**	**2.028**	**0.452**	**0.025**	**0.268**	**0.269**	**0.676**

### Statistical analysis

The number of multilocus genotypes (MLG) standardized by sample numbers, the Shannon-Wiener Index (H) (Shannon 2001), Stoddard and Taylor’s index (G) of MLG diversity (Stoddart and Taylor 1988) and Nei’s genetic distances (Nei 1978) were used for diversity analysis of the accessions. eMLG (expected MLG, an approximation of the number of genotypes that would be expected at the largest, shared sample size based on rarefaction) allows for more appropriate comparisons of the populations in case of different sample sizes. Simpson’s index (λ) (Simpson 1949) corrected by *N*/(*N*-1), evenness, E.5 (Grünwald et al. 2003), index of association (I_A_) (Brown et al. 1980; Smith et al. 1993) and Nei’s expected heterozygosity (H_Exp_) (Nei 1978) were computed for both population genetics and locus summary statistics.

MLG and Nei’s genetic distance were used for construction of a minimum spanning network. All statistical analyses were calculated using R 3.3.0 (R Core Team 2018) with poppr 2.2.0 (Kamvar et al. 2014; Kamvar et al. 2015) package.

## Results

### Development of a core SNP marker set

Thirteen accessions of chamomile (155 individual samples) were analysed with 36 SNP markers in an HRM analysis in order to obtain a better insight in the composition of varieties. Thirty-one markers of this core marker set were selected out of the 102 SNPs detected previously in the mt genome sequences of eleven chamomile accessions (Ruzicka and Novak 2020). Five markers (SNPs 90–94) were developed in addition based on a LASTZ alignment with *Diplostephium hartwegii*. One criterion for the marker selection was that only a few SNPs with rare mutations were used. All markers were checked for discriminatory power, accuracy and stability.

Mutations were verified by including previously sequenced samples in the analyses. This quality control step allowed for (i) validation of the genotyping technique, (ii) verification of the accuracy of the SNPs used for genotyping.

With the analysed dataset, we could therefore find fifteen of the originally detected mitotypes by Ruzicka and Novak (2020); five new mitotypes were detected so that in total twenty mitotypes were exhibited in this work (Online Resource 2). Mitotypes were consecutively numbered, and numbers that were found in the previous study but could not be verified here were excluded.

### Primer evaluation

In accordance with the low amount of substitutions (eleven) originally found in the coding regions of the chamomile mt genome (Ruzicka and Novak 2020), the design of only three markers (Mr_mt46728, Mr_mt154969, Mr_mt262199) for substitutions situated in coding regions was successful. In total, we screened eight transitions (6 A/G, 2 C/T) and 30 transversions (16 G/T, 12 A/C, 1 A/T, 1 G/C) with the given core marker set. One of the 36 selected SNP markers was triallelic (Mr_mt130788). With one primer combination (Mr_mt154969) two substitutions were simultaneously analysed.

ΔT_m_ between the two melting curves per locus as a score for the discriminatory power of the primers is given in Table 2. With all primer combinations ΔT_m_ values between 0.32 and 0.82 and standard deviations of 0.01 and0.05, and therefore, a distinct clustering of the melting curves could be achieved. The best resolution (ΔT_m_ 0.82) was calculated for the locus Mr_mt158868, which detects a G/T transversion. Mr_mt026887 (G/T) and Mr_mt187230 (A/C) showed the lowest ΔT_m_ values (0.32).

### Descriptive diversity statistics

**Fig. 1:**
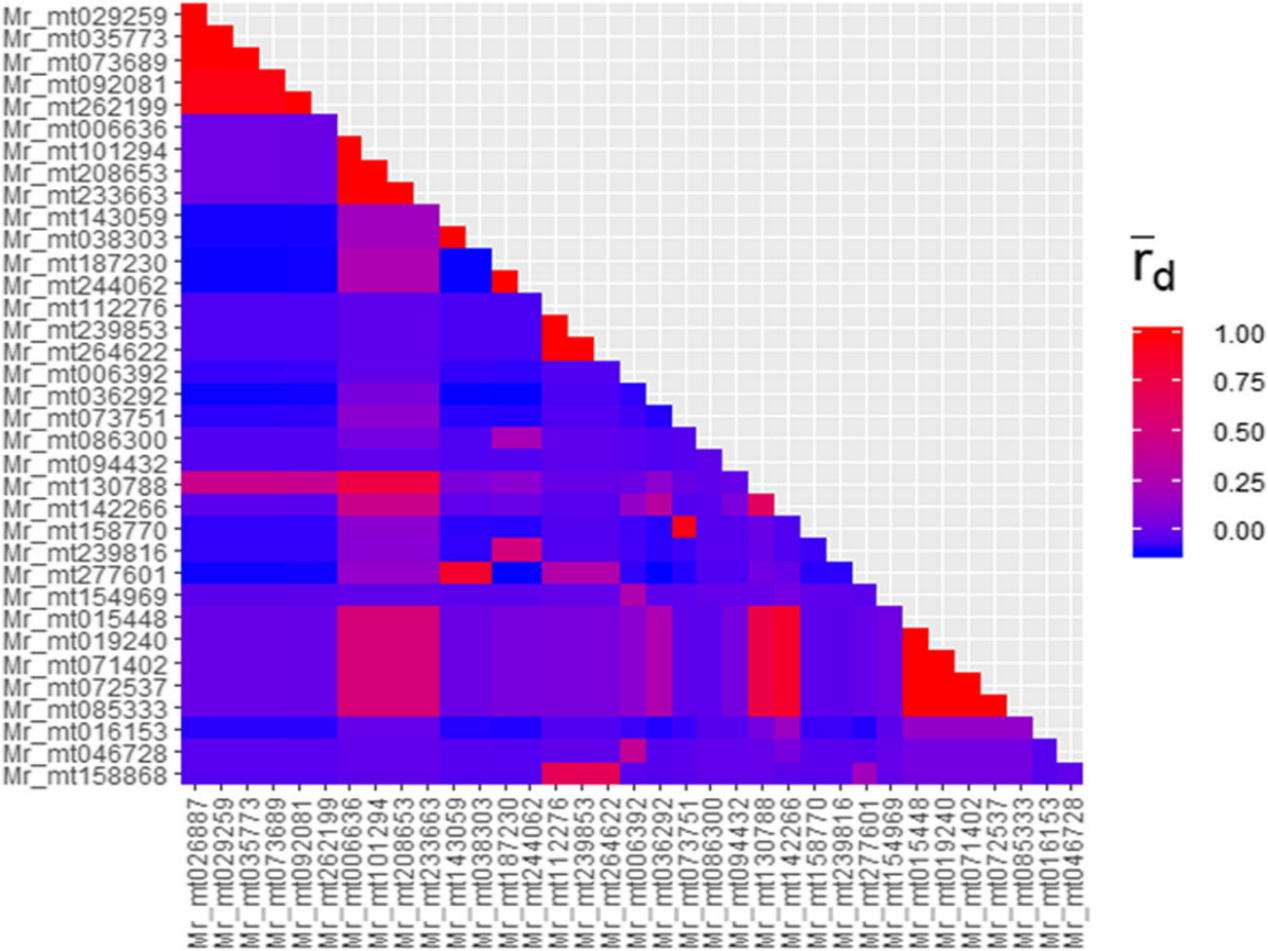
Linkage disequilibrium of the executed marker set displayed by ṝ_d_ values coloured in a gradient from blue (no correlation) to red (high correlation). The average ṝ_d_ was 0.15. In total, fifteen of the markers were strongly correlated

As expected, the triallelic marker Mr_mt130788 revealed highest values of H_exp_ and evenness and the highest Simpson’s index and therefore provided highest information content. In general, Simpson’s index ranged from 0.013 (Mr_mt154969) to 0.612 (Mr_mt130788) (mean = 0.268), the expected heterozygosity H_exp_ ranged from 0.013 to 0.616 (mean = 0.269) and evenness ranged from 0.327 to 0.985 (mean = 0.676) (Table 2). The HRM marker Mr_mt154969 was the least informative marker of this study. For estimating the linkage disequilibrium between the markers, the index of association (I_A_) was calculated across all markers. The average I_A_ was 4.79 with a *p* value of 0.001 and ṝ_d_ of 0.15 (Fig. 1). Six groups of markers comprising 5, 4, 2, 2, 3 and 5 individual markers, respectively, showed a strong linkage disequilibrium within, although these primers were not in close proximity in the mt genome. Thus, although the executed primers were chosen to be widely distributed in the alignment, 15 of the selected markers were correlated and therefore provided redundant genotyping information.

### Population genetics

**Fig. 2:**
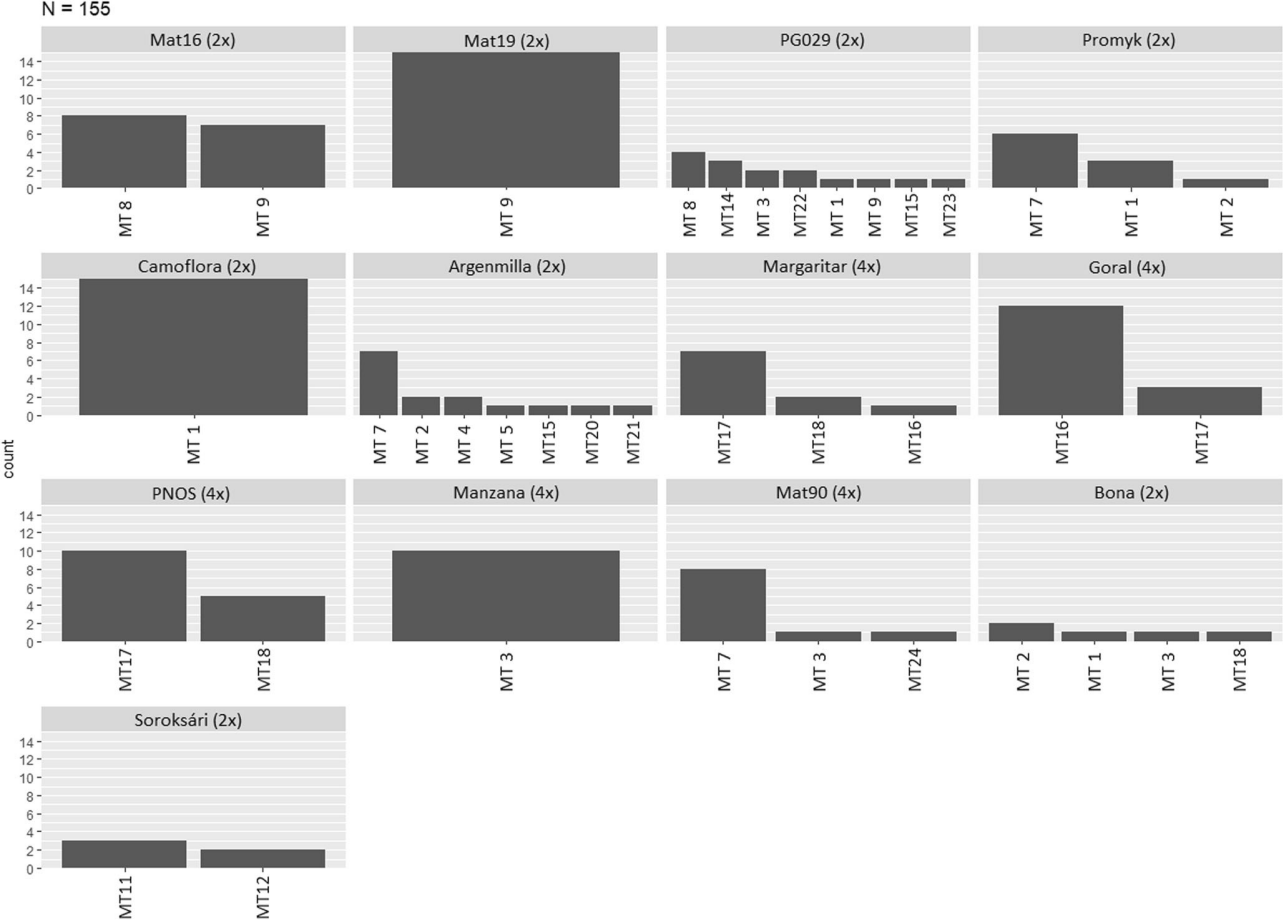
Mitochondrial haplotypes (mitotypes) identified in the given dataset of 155 chamomile samples, given are the numbers of individuals per accession that exhibit the special haplotype, (2×)/(4×) correspond to the main ploidy level (not proven in this study) of the accessions as declared by Das (2014), Otto et al. (2017) and Faehnrich et al. (2019)

Calculated AMOVA (analysis of molecular variance) showed a variability of 65.7% between the accessions and 34.3% within accessions. Thus, the mt genome of chamomile delivered a high variation for clarifying the relatedness of the accessions but also displayed a high genetic diversity within. Six of the accessions revealed an H_exp_ of > 0.1 (Table 3). With values of 0.1944 and 0.1852 ‘Margaritar’ and ‘PNOS’ showed the highest H_exp_. Nevertheless, evenness and Simpson’s index were lower for those tetraploid varieties than for the diploid accessions ‘PG029’ (E.5 0.859/λ 0.836), ‘Bona’ (E.5 0.922/λ 0.72) and ‘Argenmilla’ (E.5 0.667/ λ 0.729). Thus, the accessions with the highest genotypic richness with identified eight, seven and four MLGs (multilocus genotypes, mitotypes), respectively, were the Croatian accession ‘PG029’ (eMLG 6.45), the Argentinian ‘Argenmilla’ (eMLG 5.48) followed by ‘Bona’ from the Slovak Republic (eMLG 4) (Table 3, Fig. 2).

**Table 3:** Population genetics calculated with poppr. Given are the number of multilocus genotypes (MLG) and number of expected MLG (eMLG) at the largest sample size with standard error (SE), the Shannon-Wiener index (H) (Shannon 2001) and the Stoddart and Taylor’s index (G) (Stoddart and Taylor 1988) of MLG diversity, Simpson’s index (λ) (Simpson 1949) corrected by N/(N-1), evenness (E.5) (Grünwald et al. 2003), Nei’s expected heterozygosity (H_Exp_), index of association (I_A_) (Brown et al. 1980; Smith et al. 1993) and standardized index of association (rbarD)

Pop	*N*	MLG	eMLG	SE	*H*	*G*	*λ*	E.5	Hexp	*I*_*A*_	rbarD
Mat16	15	2	2	0	0.691	1.99	0.498	0.996	0.0889	5	1
Mat19	15	1	1	0	0	1	0	NaN	0	NaN	NaN
PG029	15	8	6.45	0.8269	1.934	6.08	0.836	0.859	0.1778	7.473	0.364
Promyk	10	3	3	0	0.898	2.17	0.54	0.807	0.0278	0.607	0.607
Camoflora	15	1	1	0	0	1	0	NaN	0	NaN	NaN
Argenmilla	15	7	5.48	0.8388	1.615	3.69	0.729	0.667	0.1553	5.422	0.241
Margaritar	10	3	3	0	0.802	1.85	0.46	0.693	0.1944	9.907	0.526
Goral	15	2	1.98	0.1466	0.5	1.47	0.32	0.725	0.1238	12	1
PNOS	15	2	2	0.0182	0.637	1.8	0.444	0.899	0.1852	13	1
Manzana	10	1	1	0	0	1	0	NaN	0	NaN	NaN
Mat90	10	3	3	0	0.639	1.52	0.34	0.576	0.0827	12.179	0.94
Bona	5	4	4	0	1.332	3.57	0.72	0.922	0.1667	9.5	0.679
Soroksári	5	2	2	0	0.673	1.92	0.48	0.961	0.05	2	1
Total	155	20	6.73	1.1316	2.496	9.64	0.896	0.777	0.2695	4.795	0.15

### Mitotype relationships

**Fig. 3:**
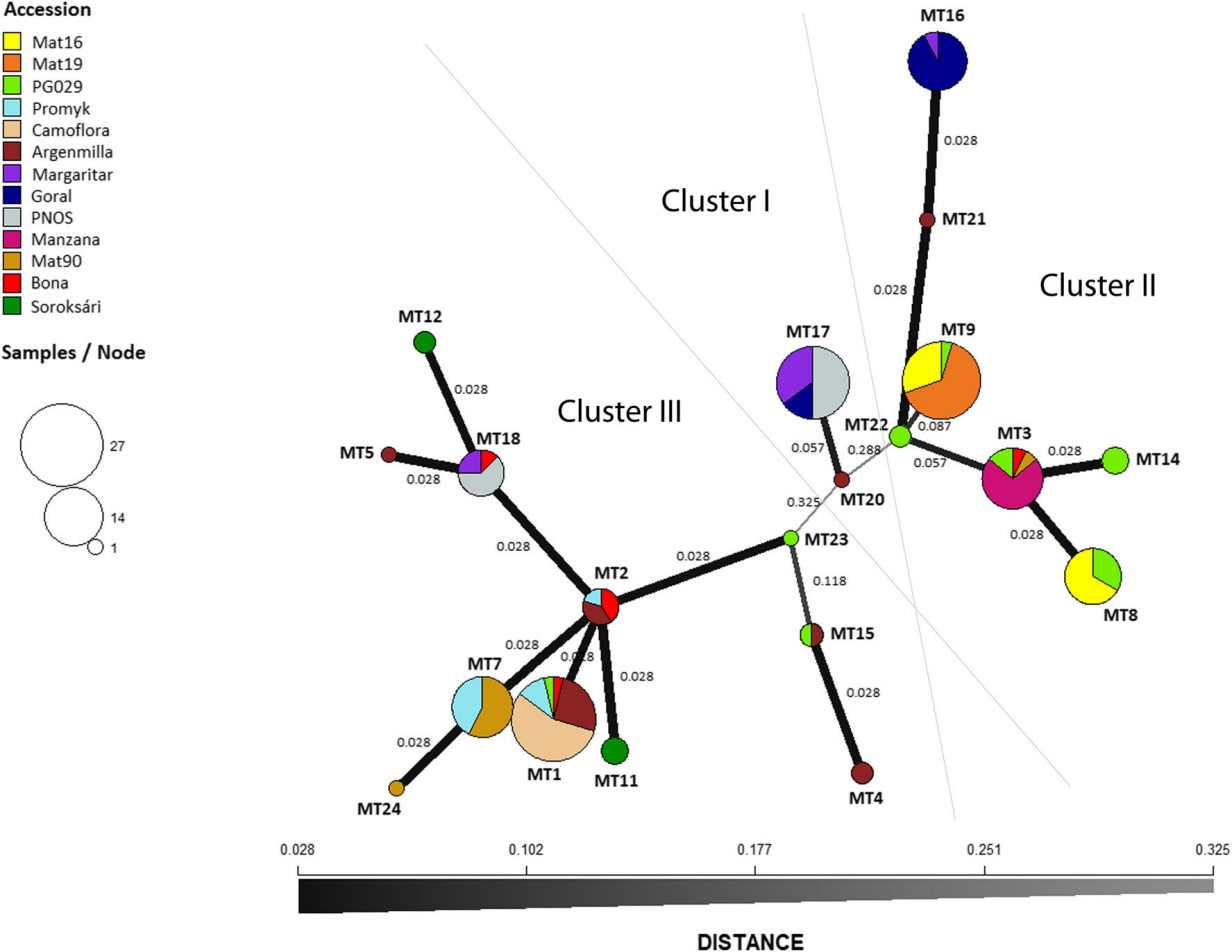
Minimum spanning network representing the identified mitotypes (MT) calculated with poppr 2.2.0 using Nei’s genetic distances. Circle areas are proportional to the number of samples per node. Genetic distances are given by different shading of a grayscale and line thicknesses corresponding to a scale bar. Values near the lines are distant values

Only three of the accessions (‘Camoflora’ (2×), ‘Mat19’ (2×) and ‘Manzana’ (4×)) were monomorphic (Fig. 2). In ten accessions, at least two different mt lineages could be found with an exceptionally high diversity in the before mentioned ‘Argenmilla’ and ‘PG029’. Genetic distances between polymorphic mitotypes were often low (0.028). The highest genetic diversity (0.693) was found between Mitotype 4 (‘Argenmilla’ 2×) and Mitotype 8 (‘Mat16’ 2×, ‘PG029’ 2×) or Mitotype 14 (‘PG029’ 2×), respectively. In the calculated minimum spanning network three main clusters with higher genetic distances (0.288–0.613) could be specified (Fig. 3). Cluster I (MT17, MT20) comprised all individuals of ‘PNOS’ (4×) and part of ‘Goral’ (4×), ‘Margaritar’ (4×) and ‘Argenmilla’ (2×). Cluster II (MT3, MT8, MT9, MT14, Mt16, MT21, MT22) consisted of ‘Mat16’ (2×), ‘Mat19’ (2×), ‘Manzana’ (4×), the other part of ‘Goral’ (4×) and ‘Margaritar’ (4×), the main part of ‘PG029’ (2×) and individuals of ‘Argenmilla’ (2×) and ‘Bona’ (2×). Cluster III (MT1, MT2, MT4, MT5, MT7, MT11, MT12, MT15, MT18, MT23, MT24) comprised the rest of the samples. Most of the accessions were spread over two of the main clusters, the accession with the highest diversity ‘Argenmilla’ and the tetraploid ‘Margaritar’ were even spread over all three clusters. Only the three monomorphic accessions as well as ‘Mat16’ (2×), ‘Promyk’ (2×) and ‘Soroksári’ (2×) were restricted to one cluster.

### Relationship between accessions

An accession-wise distance calculation reveals the greatest distances between ‘Mat19’ and the accessions ‘Promyk’ (0.522), ‘Camoflora’ (0.539) and ‘Soroksári’ (0.538), respectively (Table 4). The closest related accessions are ‘PNOS’ and ‘Margaritar’ (0.007) or ‘Mat90’ and ‘Promyk’ (0.009). The overall genetic diversity between tetraploid accessions (0.243) was only slightly higher than between diploid accessions (0.233), and the distance between diploid and tetraploid accessions was 0.252.

**Table 4:** Distance calculations between accessions based on Nei’s genetic distance (Nei 1978)

	Mat16	Mat19	PG029	Promyk	Camoflora	Argenmilla	Margaritar	Goral	PNOS	Manzana	Mat90	Bona	Soroksári
Mat16	0.0000												
Mat19	0.0498	0.0000											
PG029	0.0302	0.1164	0.0000										
Promyk	0.4783	*0.5216*^*a*^	0.3018	0.0000									
Camoflora	0.4957	*0.5390*^*a*^	0.3147	0.0241	0.0000								
Argenmilla	0.3868	0.4203	0.2335	0.0233	0.0189	0.0000							
Margaritar	0.3217	0.3650	0.2388	0.2760	0.2927	0.2161	0.0000						
Goral	0.1046	0.1480	0.0888	0.4139	0.4311	0.3271	0.1810	0.0000					
PNOS	0.3696	0.4129	0.2634	0.2140	0.2305	0.1678	*0.0069*^*b*^	0.2327	0.0000				
Manzana	0.0397	0.1495	0.0229	0.4753	0.4925	0.3932	0.3219	0.1143	0.3677	0.0000			
Mat90	0.4008	0.4538	0.2484	*0.0094*^*b*^	0.0575	0.0414	0.2549	0.3549	0.2035	0.3921	0.0000		
Bona	0.2979	0.3592	0.1652	0.0267	0.0338	0.0162	0.2074	0.2709	0.1641	0.2851	0.0316	0.0000	
Soroksári	0.4945	*0.5378*^*a*^	0.3168	0.0330	0.0486	0.0384	0.2822	0.4290	0.2153	0.4904	0.0494	0.0333	0.0000

^a^ Highest genetic distance values, ^b^ Lowest genetic distance values

### Distribution of the mitotypes

**Fig. 4:**
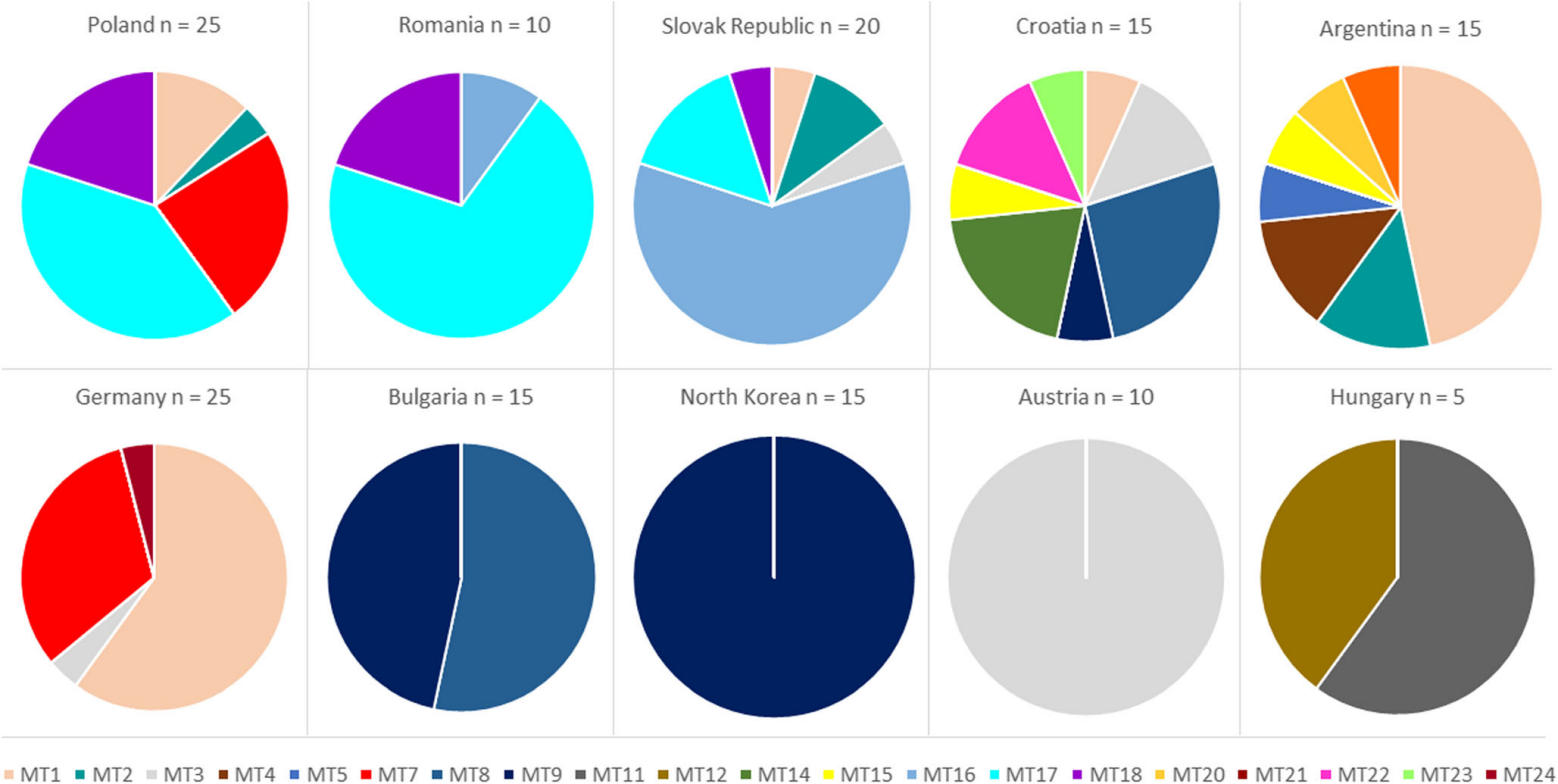
Pie graph displaying the distribution of the mitotypes (MT) in geographical origins and total number of individuals per country

All of the mitotypes were distributed over several countries despite of the ten mitotypes represented by single individuals of one accession only (MT4, MT5, MT11, MT12, MT14, MT20, MT21, MT22, MT23, MT24). The mitotypes with the highest frequency in chamomile were MT1, MT9 and MT17 (Fig. 3, Fig. 4). Only one mitotype each was detected in the Austrian and North Korean accession (Fig. 4). Croatia and Argentina retrieve the highest variability caused by the two heterogeneous accessions ‘PG029’ and ‘Argenmilla’. Anyhow, those two were the accessions with the most infrequent mitotypes. The small group of Hungarian samples was split into two mitotypes, which were not present in any other country. The other countries often formed groups of three with two to three identical frequently occurring lines on average. Bulgaria held two mitotypes; one of them also occurred in Croatia, the other one was identical to the North Korean accession. The two main mitotypes of Poland and Romania were MT17 and MT18. Both were in smaller proportion also present in the Slovak Republic. Although the Slovak Republic comprised six mt lineages, MT16 was the mitotype with the highest prevalence in this country. This mitotype could also be found in Romania, so that Poland, Romania and the Slovak Republic shared three to four mitotypes. The main mitotypes of Germany were MT1 and MT7 equally existing in Poland. MT1, in lower frequency also present in two further countries, was the mitotype with the highest frequency in the Argentinian accession. The ‘Austrian’ MT3 was distributed in the Slovak Republic, Croatia and Germany, thus building another triad.

In general, it seems that frequent mitotypes of chamomile are distributed in most cases over two to three countries.

## Discussion

### Comparison to a previous mt chamomile study

Twenty mitotypes could be specified with a core marker set of 36 HRM markers in the mt genome of chamomile. In a previous study nineteen mitotypes were identified in the mt genome of 33 individuals by NGS (Ruzicka and Novak 2020). Four of these previously sequenced mitotypes could not be verified via HRM. Five mitotypes (MT20–24) were additionally found in the larger sample set, so that at least twenty-four mitotypes coexist in *M. chamomilla*. The use of an extended sample set corroborated the mt heterogeneity of the accessions ‘PG029’ and ‘Argenmilla’ whereas ‘Camoflora’, the only monomorphic accession of the first investigation (Ruzicka and Novak 2020), remained monomorphic. Other than previously assumed, identical mitotypes within di- and tetraploid accessions existed, Mitotype 3, e.g. with highest proportion of ‘Manzana’, was also present in ‘Bona’, ‘PG029’ and ‘Mat90’. Furthermore, the accession ‘Mat19’ appeared monomorphic in this study, because no suitable primer could be designed for the underlying mutation while two mitotypes were found in this accession in the previous study (Ruzicka and Novak 2020). In both studies, three main clusters could be found with more or less identical groups of mitotypes within these clusters.

### Mitochondrial diversity examined in this study

As we examined substitutions only, the likelihood that an even higher amount of total mt variation in *M. chamomilla* is present is high. Plant mt DNA normally shows frequent recombination but a poor substitution rate (Gualberto et al. 2014). Therefore, this high number of maternally inherited point mutations in the mt DNA we found in closely related chamomile accessions is astonishing. An equal proportion of point mutations in the mt genome of other Asteraceae below the interspecific level has not yet been documented (Ruzicka and Novak 2020).

The hitherto examined genetic similarity of cultivated chamomile based on nuclear polymorphisms was high (Wagner et al. 2005; Okon et al. 2013; Otto et al. 2017). It is not unusual that the nuclear similarity was lower the more wild populations or geographically distinct populations were included in the analysis. Therefore, Wagner et al. (2005) and Okon et al. (2013) detected higher genetic similarities than Solouki et al. (2008), who compared European and Iranian accessions. Nevertheless, Otto et al. (2017) determined higher genetic similarity especially of tetraploid accessions but without clear geographical correlation. The mt variability found in this study is comparable to nuclear variability of chamomile from the same region, namely Central-Europe (Wagner et al. 2005; Okon et al. 2013). In fact, high mt variability in this study was not only detected between but also within the accessions. Consecutive selection and isolation of the breeding material will most likely be responsible for the increasing homogeneity of nuclear germplasm of cultivated chamomile. Although uniparentally inherited genome evolves more slowly than nuclear genome it is obvious that despite of diminished diversity of the nuclear genome, the mt DNA of cultivated chamomile reflects a higher variability.

### Possible relations between mitotype distribution and development of chamomile varieties

Ten of the examined accessions are polymorphic with at least two mitotypes. In two accessions even seven (‘Argenmilla’), respectively nine (‘PG029’) mitotypes were identified though most of them with low frequencies. Although the results might be biased by different sample volumes in the countries (e.g. two accessions in Germany, Poland and Slovak Republic versus one accession in the other countries, only five individuals from Hungary), it is obvious that Croatia and Argentina retrieve the highest variability caused by these two accessions.

Several hypotheses are possible to explain the high number of point mutations found within accessions of chamomile: (I) spontaneous mutations, which would explain the low diversity found between some of the mitotypes; (II) use of several plant lines to produce cultivars or traded landraces; (III) exchange of plant material between breeders to establish new chamomile lines in other countries; (IV) cultivation of imported chamomile plants in historical times; (V) migration of chamomile populations into several countries and establishment of those chamomile populations with identical mother lines, which were independently used for producing new cultivars; (VI) contamination of seed material of cultivated chamomile with seeds of wild growing chamomile.

The fact that most of the accessions are divided into two or three main mitotypes with high frequencies corroborates the hypotheses of several promising lineages that were used for producing the cultivars and landraces (thinkable for e.g. ‘Soroksári’ and ‘Mat16’) or the extensive exchange of plant material between breeders (e.g. Romanian and Polish accessions). It is known that the Romanian ‘Margaritar’ originates from the Polish ‘Zloty Lan’ and chamomile plants collected in the wild in Poland (Das 2014). This could explain the close relationship between ‘Margaritar’ and ‘PNOS’ we found in our study. The low geographical distance between Polish and German breeding centres in the past could also result in identical mitotypes in different cultivars due to identical cytoplasmic lineages of the underlying natural populations. Nevertheless, according to Das (2014), ‘Bona’ and ‘Goral’ should be progenies of breeding experiments using the same plant material, but a close genetic relationship of those accessions cannot be confirmed by our data. If the North Korean accession ‘Mat19’ would have been the result of a chamomile population arisen independently in North Korea, we would have expected it to be more distantly related to the other accessions. Here again, the use of breeding material from other countries (e.g. Bulgaria or Croatia) or even the import and establishment of chamomile cultivation in ancient times is supposable.

The high number of monotypic mitotypes in the accessions ‘PG029’ and ‘Argenmilla’ could better be explained by contamination of the seed material or a higher tendency of spontaneous point mutations in these accessions than by extensive plant exchange alone. Nevertheless, ‘Argenmilla’ and ‘PG029’ also were more distinct to many of the other chamomile accessions in the GBS study of Otto et al. (2017).

### Applicability for detecting a CMS phenotype

An increased mt mutation rate was detected in an infertile line of onion, where it co-occurs with CMS (Kim et al. 2016; Kim and Kim 2019). Crossings of highly variable chamomile lines or of distantly related accessions might enhance the possibility to find CMS in the progenies. The high amount of substitutions within the accessions ‘Argenmilla’ (2×) and ‘PG029’ (2×) are promising to induce negative interactions of the mt and nuclear genome often resulting in the occurrence of a CMS phenotype. Although distance calculations identify ‘Mat19’ (2×) and ‘Camoflora’ (2×) or ‘Soroksári’ (2×) as the most distantly related and therefore the most adequate candidates for inter-cultivar crossings, the high diversity of the examined accessions will necessitate the genetic analysis of individuals, especially when highly polymorphic accessions as ‘Argenmilla’ and ‘PG029’ should be included.

## Conclusions

Our results confirm the high extent of variations found in a previous study in the mt genome of chamomile and indicate that distant inter-cultivar crossings with the aim to exhibit a CMS phenotype in the progenies could be successful. However, it is obvious that an accession-based analysis might not be sufficient but that an individual-based screening will have to be executed prior to crossing trials. HRM analysis proved to be a valuable tool for the screening of point mutations in a high amount of samples and could therefore serve as adequate facility for this purpose.

Several hypotheses are possible to explain the occurrence of multiple mitotypes per accession in chamomile. The additional analysis of natural populations would be necessary to prove or exclude some of the hypotheses presented here. However, based on this dataset, a multi-causal source seems to be most likely.

## Supplementary information

Online Resource 1 List of the primer pairs used in this study. Given are the primer names corresponding to the position of the mutation in the alignment, the detected SNP and amplicon length, annealing temperature and HRM ramp (the number in the primer name corresponds to the position in the LASTZ-Alignment) (XLSX 12 kb)

Online Resource 2 Polymorphic sites investigated with HRM analysis, alignment position corresponding to sequence coordinates of *Diplostephium hartwegii*, individual sample names and the resulting definition of mitotypes (MT). SNPs and MT are consecutively named according to a previous study (Ruzicka and Novak 2020), SNPs 90-94 are new markers examined in this study and integrated according to their position in the alignment; MT6, MT10, MT13 and MT19 are missing as they could not be discriminated in this study from MT1, MT9, MT8 and MT17, respectively (XLSX 1681 kb)

## Data Availability

NGS sequence information is available on NCBI (National Center for Biotechnology Information, https://www.ncbi.nlm.nih.gov/), Submission ID: SUB5046906/ BioProject ID: PRJNA515664. Substitutions of the examined dataset are presented in Online Resource 2.

## References

[CR1] Ahmadi H, Rahimmalek M, Zeinali H (2014). Assessment of the genetic variation of chamomile (Matricaria chamomilla L.) populations using phytochemical, morphological and ISSR markers. Biochem Syst Ecol.

[CR2] Albrecht S, Sonnenschein M, Plescher A (2016) Breeding of a high yielding chamomile variety (*Matricaria recutita* L.) with improved traits for machine harvesting. Julius-Kühn-Archiv 453. 10.5073/jka.2016.453.048

[CR3] Arroyo-García R, Ruiz-García L, Bolling L, Ocete R, López MA, Arnold C (2006). Multiple origins of cultivated grapevine (*Vitis vinifera* L. ssp. *sativa*) based on chloroplast DNA polymorphisms. Mol Ecol.

[CR4] Balk J, Leaver CJ (2001). The PET1-CMS mitochondrial mutation in sunflower is associated with premature programmed cell death and cytochorome c release. Plant Cell.

[CR5] Borna T, Salami SA, Shokrpour M (2017). High resolution melting curve analysis revealed SNPs in major cannabinoid genes associated with drug and non-drug types of cannabis. Biotechnol Biotechnol Equip.

[CR6] Brown AHD, Feldman MW, Nevo E (1980) Multilocus structure of natural populations of *Hordeum spontaneum*. Genetics 96:523–536. http://www.genetics.org/content/96/2/523. Accessed July 2019 10.1093/genetics/96.2.523 PMC1214315 17249067

[CR7] Budar F, Touzet P, De Paepe R (2003). The nucleo-mitochondrial conflict in cytoplasmic male sterilities revisited. Genetica.

[CR8] Bustin SA, Benes V, Garson JA, Hellemans J, Huggett J, Kubista M (2009). Clin Chem.

[CR9] Das M (2014). Chamomile. Medicinal, biochemical, and agricultural aspects.

[CR10] Faehnrich B, Nemaz P, Franz C (2013). Self-incompatibility and male sterility in six *Matricaria recutita* varieties. J Appl Bot Food Qual.

[CR11] Faehnrich B, Otto L-G, Franz C, Mesic E, Cosendai A-C, Dobes C (2019). Auxin application in interploidy crosses and genome stability: across-generation investigations on German chamomile (*Matricaria recutita* [L.] Rauschert) of various origins. Euphytica.

[CR12] Franke R, Schilcher H (2007). Relevance and use of chamomile (*Matricaria recutita* L.). ISHS. Acta Hortic.

[CR13] Germplasm Resources Information Network (GRIN) (2019) Beltsville (MD): United States Department of Agriculture, Agricultural Research Service.https://npgsweb.ars-grin.gov/gringlobal/taxonomydetail.aspx?id=23475. Accessed 01 Jul 2019

[CR14] Grünwald NJ, Goodwin SB, Milgroom MG, Fry WE (2003) Analysis of genotypic diversity data for populations of microorganisms. Phytopathology 93:738–746. http://apsjournals.apsnet.org/doi/abs/10.1094/PHYTO.2003.93.6.738. Accessed July 2019 10.1094/PHYTO.2003.93.6.738 18943061

[CR15] Gualberto JM, Mileshina D, WalletC NAK, Weber-Lotfi F, Dietrich A (2014). The plant mitochondrial genome: dynamics and maintenance. Biochimie.

[CR16] Gugerli F, Sperisen C, Buchler U, Magni F, Geburek T, Jeandroz S, Senn J (2001). Haplotype variation in a mitochondrial tandem repeat of Norway spruce (*Picea abies*) populations suggests a serious founder effect during postglacial re-colonization of the western Alps. Mol Ecol.

[CR17] Gupta V, Mittal P, Bansal P, Khokra SL, Kaushik D (2010). Pharmacological potential of *Matricaria recutita* - a review. Int J Pharmaceut Sci Drug Res.

[CR18] Kamvar ZN, Tabima JF, Grünwald NJ (2014). Poppr: an R package for genetic analysis of populations with clonal, partially clonal, and/or sexual reproduction. Peer J.

[CR19] Kamvar ZN, Brooks JC, Grünwald NJ (2015). Novel R tools for analysis of genome-wide population genetic data with emphasis on clonality. Front Genet.

[CR20] Kearse M, Moir R, Wilson A, Stones-Havas S, Cheung M, Sturrock S (2012). Geneious Basic: an integrated and extendable desktop software platform for the organization and analysis of sequence data. Bioinformatics.

[CR21] Kim B, Kim K, Yang TJ, Kim S (2016). Completion of the mitochondrial genome sequence of onion (*Allium cepa* L.) containing the CMS-S male-sterile cytoplasm and identification of an independent event of the ccmF N gene split. Curr Genet.

[CR22] Kim B, Kim S (2019). Development of molecular markers for distinguishing onion (*Allium cepa* L.) and Welsh onion (*A*. *fistulosum* L.) based on polymorphic mitochondrial genome sequences. Plant Breed Biotech.

[CR23] Koressaar T, Remm M (2007). Enhancements and modifications of primer design program Primer3. Bioinformatics.

[CR24] Liu Z, Cai X, Seiler G, Jan C (2014). Interspecific amphiploid-derived alloplasmic male sterility with defective anthers, narrow disc florets and small ray flowers in sunflower. Plant Breed.

[CR25] Liu Z, Wang D, Feng J, Seiler G, Cai X, Jan C (2013). Diversifying sunflower germplasm by integration and mapping of a novel male fertility restoration gene. Genetics.

[CR26] Nei M (1978) Estimation of average heterozygosity and genetic distance from a small number of individuals. Genetics 89:583–590. http://www.genetics.org/content/89/3/583.abstract. Accessed July 2019 10.1093/genetics/89.3.583 PMC1213855 17248844

[CR27] Okon S, Surmacz-Magdziak A, Paczos-Grzeda E (2013). Genetic diversity among cultivated and wild chamomile germplasm based on ISSR analysis. Acta Sci Pol Hortorum Cultus.

[CR28] Otto L-G, Mondal P, Brassac J, Preiss S, Degenhardt J, He S (2017). Use of genotyping-by-sequencing to determine the genetic structure in the medicinal plant chamomile, and to identify flowering time and alpha-bisabolol associated SNP-loci by genome-wide association mapping. BMC Genomics.

[CR29] Pirkhezri M, Hassani ME, Hadian J (2010). Genetic diversity in different populations of Matricaria chamomilla L.growing in southwest of Iran, based on morphological and RAPD markers. Res J Med Plant.

[CR30] R Core Team (2018) R: a language and environment for statistical computing. Hg. v. R Foundation for Statistical Computing, ViennaOnline. https://www.R-project.org/. Accessed July 2019

[CR31] Ruzicka J, Novak J (2020). Mitochondrial genome variation between different accessions of *Matricaria chamomilla* L. (Asteraceae) based on SNP mutation analysis. Genet Resour Crop Evol.

[CR32] Schmiderer C, Lukas B, Novak J (2013). Effect of different DNA extraction methods and DNA dilutions on the amplification success in the PCR of different medicinal and aromatic plants. Z Arznei- Gewurzpfla.

[CR33] Seidler-Lozykowska K (2016) Medicinal plant breeding in Poland: history and nowadays, in Proceedings of the 6th International Symposium Breeding Research on Medicinal and Aromatic Plants, BREEDMAP 6, Quedlinburg, Germany 10.5073/jka.2016.453.023

[CR34] Shannon CE (2001) A mathematical theory of communication. ACM SIGMOBILE Mobile Comput Commun Rev 5:3–55. http://cm.bell-labs.com/cm/ms/what/shannonday/shannon1948.pdf. Accessed July 2019

[CR35] Simpson EH (1949). Measurement of diversity. Nature.

[CR36] Smith JM, Smith NH, O’Rourke M, Spratt BG (1993) How clonal are bacteria. Proc Natl Acad Sci 90:4384–4388. http://www.pnas.org/content/90/10/4384. Accessed July 2019 10.1073/pnas.90.10.4384 PMC46515 8506277

[CR37] Solouki M, Mehdikhani H, Zeinali H, Emamjomeh AA (2008). Study of genetic diversity in chamomile (*Matricaria chamomilla*) based on morphological traits and molecular markers. Sci Hortic.

[CR38] Sorkheh K, Dehkordi MK, Ercisli S, Hegedus A, Halász J (2017). Comparison of traditional and new generation DNA markers declares high genetic diversity and differentiated population structure of wild almond species. Sci Rep.

[CR39] Spassova M, John H, Nijkamp J, Hille J (1993). Cytoplasmic sterility in higher plants. Biotechnol Biotechnol Equip.

[CR40] Stoddart JA, Taylor JF (1988) Genotypic diversity: estimation and prediction in samples. Genetics 118:705–711. http://www.genetics.org/content/118/4/705. Accessed July 2019 10.1093/genetics/118.4.705 PMC1203325 17246421

[CR41] The Plant List (2013) Version 1.1. http://www.theplantlist.org/tpl1.1/record/gcc-103038. Accessed 01 July 2019

[CR42] Tomaru N, Takahashi M, Tsumura Y, Takahashi M, Ohba K (1998). Intraspecific variation and phylogeographic patterns of *Fagus crenata* (Fagaceae) mitochondrial DNA. Am J Bot.

[CR43] Untergasser A, Cutcutache I, Koressaar T, Ye J, Faircloth BC, Remm M, Rozen SG (2012). Primer3—new capabilities and interfaces. Nucleic Acids Res.

[CR44] Wagner C, Friedt W, Marquard RA, Ordon F (2005). Molecular analyses on the genetic diversity and inheritance of (-)-abisabolol and chamazulene content in tetraploid chamomile (*Chamomilla recutita* (L.) Rausch.). Plant Sci.

